# APN Expression in Serum and Corpus Luteum: Regulation of Luteal Steroidogenesis Is Possibly Dependent on the AdipoR2/AMPK Pathway in Goats

**DOI:** 10.3390/cells12101393

**Published:** 2023-05-15

**Authors:** Xiaomeng Pei, Haolin Li, Hao Yu, Wei Wang, Dagan Mao

**Affiliations:** College of Animal Science and Technology, Nanjing Agricultural University, Nanjing 210095, China; 2019205010@njau.edu.cn (X.P.); lihaolin504@163.com (H.L.); 2020205009@stu.njau.edu.cn (H.Y.);

**Keywords:** APN, AdipoR, T-Ca, AdipoRon, P-AMPK, steroidogenesis

## Abstract

Adiponectin (APN) is an essential adipokine for a variety of reproductive processes. To investigate the role of APN in goat corpora lutea (CLs), CLs and sera from different luteal phases were collected for analysis. The results showed that the APN structure and content had no significant divergence in different luteal phases both in CLs and sera; however, high molecular weight APN was dominant in serum, while low molecular weight APN was more present in CLs. The luteal expression of both AdipoR1/2 and T-cadherin (T-Ca) increased on D11 and 17. APN and its receptors (AdipoR1/2 and T-Ca) were mainly expressed in goat luteal steroidogenic cells. The steroidogenesis and APN structure in pregnant CLs had a similar model as in the mid-cycle CLs. To further explore the effects and mechanisms of APN in CLs, steroidogenic cells from pregnant CLs were isolated to detect the AMPK-mediated pathway by the activation of APN (AdipoRon) and knockdown of APN receptors. The results revealed that P-AMPK in goat luteal cells increased after incubation with APN (1 μg/mL) or AdipoRon (25 μM) for 1 h, and progesterone (P4) and steroidogenic proteins levels (STAR/CYP11A1/HSD3B) decreased after 24 h. APN did not affect the steroidogenic protein expression when cells were pretreated with Compound C or SiAMPK. APN increased P-AMPK and reduced the CYP11A1 expression and P4 levels when cells were pretreated with SiAdipoR1 or SiT-Ca, while APN failed to affect P-AMPK, the CYP11A1 expression or the P4 levels when pretreated with SiAdipoR2. Therefore, the different structural forms of APN in CLs and sera may possess distinct functions; APN might regulate luteal steroidogenesis through AdipoR2 which is most likely dependent on AMPK.

## 1. Introduction

After ovulation, the inner part of the mammalian ovary forms a temporary endocrine gland known as the corpus luteum (CL), which is formed by numerous cell classes, e.g., steroidogenic cells, immune cells, fibroblasts, pericytes and endothelial cells [1,2,3,4]. The lifecycle of the CL comprises three major stages: formation, maturation and regression [5]. During maturation, the CL is able to produce progesterone (P4), which regulates the estrous cycle and maintains pregnancy. By the end of the pregnancy or in the absence of a fertilization signal, the CL gradually regresses [6]. Extensive evidence has proven that the CL requires a system coordinating ovarian steroid hormones which is regulated by steroidogenic acute regulatory protein (STAR), cytochrome P450 cholesterol side-chain cleavage enzyme (P450scc/CYP11A1) and 3β-hydroxysteroid dehydrogenase (HSD3B) [4,7,8].

Cytokines and chemokines are closely related to animal reproduction. Previous studies have identified strong links between adiponectin (APN, also known as ACRP30, GBP28, apM1 and adipoQ) and reproduction, including steroidogenesis [9], folliculogenesis [10], oocyte maturation [11], luteal function [12] and luteolysis [13]. A higher postpartum serum APN concentration indicates a normal and earlier onset of luteal activity in cows with a high milk yield [14], and a high association of the APN gene with the number of litters has been found in Awassi ewes [15]. Acute upregulation of the APN system is more likely related to food-deprivation-induced damage of mouse ovaries [16], and food restriction enhances the expression of the APN system in pre-pubertal ewe ovaries, accompanied by reduced serum concentrations of steroid hormones [17], indicating that APN affects reproductive functions. APN and AdipoR1/2 are expressed in the CL of several species. APN reduces P4 secretion in cultured bovine luteal cells [12]. However, how APN affects the luteal function in goats is not yet known.

APN is secreted by white adipose tissues, and the circulating APN concentration is substantially higher than other cytokines and hormones. It is secreted in the form of a post-translation modification, including low molecular weight (LMW) trimers, middle molecular weight (MMW) hexamers, high molecular weight (HMW) multimers and the 18–25 kDa globular fragment which is produced and secreted by proteolytic cleavage of the LMW trimers [18,19,20]. However, the structure of APN in goat serum and the CL is not known.

The biological effects of APN are performed at at least three receptors: AdipoR1, AdipoR2 and T-Ca. Both AdipoR1 and AdipoR2 possess seven helices opposite to G-protein-coupled receptors and enhance the activities of adenosine monophosphate-activated protein kinase (AMPK) and peroxisome proliferator-activated receptor (PPAR), respectively. T-Ca contains glycosyl phosphatidylinositol-anchored cadherin and activates intracellular calcium and the AMPK signal [21,22,23,24]. Several studies have identified the expression and localization of APN and AdipoR1/2 in mammalian ovaries [11,16,25]. Moreover, HMW APN evokes intracellular AMPK signal transmission via conjugation to T-Ca and manages T-Ca related glucose tolerance [26,27,28]. These findings suggest that the APN system may transmit regulatory signals to the normal female reproductive function via AMPK.

AMPK serves as an antenna for cellular energy homeostasis and regulates key metabolic processes. AMPK activity is switched on by a high intracellular AMP/ATP ratio, which facilitates phospho-Thr172 AMPK and is accompanied by a cease in energy and glucose homeostasis through Akt/AMPK and AMPK/PGC1-α [29,30,31,32]. Activation of AMPK has been demonstrated to promote catabolism and suppress anabolism. Adaptor protein, phosphotyrosine interacting with PH domain and leucine zipper 1 (APPL1) and liver kinase B1 (LKB1) are the two main kinases responsible for activating AMPK. The activation of APPL1 in the APN system is essential for LKB1 cytosolic translocation [33], which leads to a diminished ATP consumption and fatty acid and glucose metabolism and to an accelerated ATP production of inactivated mTOR and glucose transport [34,35,36,37,38]. Activation of AMPK via AICAR reduces P4 levels in rat granulosa cells [33] and diminishes the luteinizing hormone-facilitated P4 secretion of bovine luteal cells [39]. Moreover, in bovine granulosa and luteal cells, metformin decreases P4 secretion through the AMPK pathway [40,41]. Thus, activation of AMPK has a negative effect on ovarian steroidogenesis in different species.

Although the effects of APN and AMPK on P4 synthesis have been clearly elucidated [40,42,43,44], the molecular mechanism underlying the function of APN/AMPK signals in goat CL has not investigated. We speculated that circulating APN and the APN system in the ovaries are significant factors for the coordination of luteal functions in goats. This study intends to identify the expression patterns of serum and luteal APN and the functions of the APN/AMPK system in the modulation of P4 synthesis in the goat CL.

## 2. Materials and Methods

### 2.1. Animals

Twelve female Haimen goats aged 2–3 years were obtained from Haimen Goat Research Center (Nantong, Jiangsu, China). Goats were estrous synchronized with a two-time PGF_2a_ treatment. In the next natural estrous cycle, goat ovaries and sera were acquired in the early (D4), mid (D11) or late (D17) luteal phase (D0 was considered as onset of estrus, *n* = 3) [12,45,46]. Sera were also collected on D0.

Goats were starved for 18–22 h prior to tissue and sera collection by a surgical approach [47]. CLs were separated for paraffin embedding and protein extraction.

To investigate the mechanism of APN-induced steroidogenic dysfunction, luteal cells were isolated from ovarian CL. Due to the similar cellular structure and function of the pregnant CL to the mid-cycle CL, ovaries were acquired from healthy pregnant goats at the early gestational stage after slaughter at an abattoir (Zhenjiang, Jiangsu, China). The duration of gestation was calculated from the embryo body weight (50 ± 20 g) [48,49]. Goat ovaries were brought to the laboratory with cold physiological saline.

### 2.2. Luteal Cell Isolation

Luteal cells were isolated as previously described [39]. CLs were separated and rinsed extensively with cold PBS containing antibiotics (100 U of penicillin and 100 mg/mL of streptomycin) to remove the blood. Then, CLs were scissor minced on ice and digested with collagenase II solution (2 mg/mL, Sigma, St. Louis, MO, USA) in DMEM/F12 (DF12, Corning, NY, USA) medium containing 0.005% DNase I and 0.5% BSA for 30 min in a water bath (37 °C). The supernatant was processed by a 200 mesh filter to remove large matrix debris and then centrifuged and resuspended in DF12 containing 10% fetal bovine serum (FBS, Corning, NY, USA) with antibiotics and cultured at 37 °C in 5% CO_2_. The medium was changed every 3 to 4 days and non-adherent cells were removed by replacement of the medium.

### 2.3. Luteal Cell Culture

Luteal cells were cultured (5% CO_2_, 37 °C) in a medium containing 10% FBS with antibiotics. The next day, cells were cultured overnight in DF12. On the day of the experiment, cells were cultured with DF12 for 2–3 h prior to medicine administration as described in the figures [50]. Cells were treated with APN (Sangon, Shanghai, China) [12], AdipoRon (MedChemExpress, Monmouth Junction, NJ, USA) [51] or AMPK antagonist (Compound C, Apexbio, Houston, TX, USA) [52,53]. The doses and pretreatment times are given in the figures; they were 1 μg/mL APN or 25 μM AdipoRon for 1 h or 24 h and 10 μM Compound C for 1 h.

### 2.4. Western Blotting

CLs and luteal cells were lysed using whole cell contained protease and phosphatase inhibitor (Solarbio, Beijing, China), separately. Cytoplasmic proteins were extracted with a cytosolic protein extraction kit (Beyotime Biotechnology, Shanghai, China). For whole cell lysis, after 20 min incubation on ice, cells were centrifuged. For cytoplasmic lysis, after 15 min incubation on ice, cells were centrifuged. Supernatants were detected by SDS-PAGE.

Standard SDS-PAGE was used for complete reduction of the sample. The 2× sample buffer for reducing conditions included 4% SDS, 120 mM Tris-HCl pH 6.8, 10% 2-mercaptoethanol, 20% glycerol and 0.06% bromophenol blue. Samples with loading buffer were heated [54]. Non-reducing conditions were employed to separate multimer forms of APN, and the 2-mercaptoethanol was removed from 2× sample buffer [18,55].

The proteins were then transferred to polyvinylidene difluoride membranes. Membranes were blocked with 1× TBST containing 5% BSA for 1 h. Primary antibodies (APN, T-Ca, LKB1, APPL1, P-AMPK, AMPK, AdipoR1, AdipoR2, CYP11A1, HSD3B, STAR (Table 1)) were incubated overnight at 4 °C. GAPDH was designated as the internal control. Secondary antibodies were incubated for 1 h at room temperature. Subsequently, the blots were visualized using ECL regents (Vazyme, Nanjing, China) and imaged using ChemiDoc (Bio-Rad, Hercules, CA, USA).

### 2.5. Multiplex Immunohistochemistry/Immunocytochemistry

Multiplex immunohistochemistry/immunocytochemistry (mIHC/mICC) was performed as previously described [56]. Paraffin-embedded luteal sections were first dewaxed and subjected to microwave treatment for antigen retrieval. The luteal cells were seeded on glass coverslips (Solarbio, Beijing, China). The endogenous peroxidase activity was quenched by hydrogen peroxide. Slides were then blocked and incubated with primary antibody (Table 1, APN, T-Ca, AdipoR1, AdipoR2, P-AMPK, AMPK, HSD3B, STAR) at 4 °C overnight. The negative controls (NC) were established by replacing the primary antibody with normal serum [57]. Sections were further incubated with a standard streptavidin–biotin–peroxidase complex (Boster Biological Technology, Wuhan, China) at room temperature. Then, a chromogenic working solution was added and optimized by employing a triplex (AF488, Cy3 and Cy5, ATT, Dallas, TX, USA) and were finished with DAPI. Image capture was performed with a Zeiss LSM900 (Zeiss, Jena, Germany).

### 2.6. RNA Silencing

For RNA interference, small interfering RNAs (SiRNAs) (SiAMPK F-AAUAAUGAACCAAGCCAGUGAGU/R-ACUCACUGGCUUGGUUCAUUAUU; SiAdipoR1 F-GGCUGAAGGACAAUGACUATT/R-UAGUCAUUGUCCUUCAGCCTT; SiAdipoR2 F-GGAUAAUGACUUCCUCCUATT/R-UAGGAGGAAGUCAUUAUCCTT; SiT-Ca F-CAUACUUCAAGGUGAACAATT/R-UUGUUCACCUUGAAGUAUGTT; Scrambled non-targeting RNA F-AAAA/R-UUUU) were purchased from Tsingke (China). Scrambled non-targeting RNA was transfected into cells as a negative control. Cells were transfected with 100 μM SiRNA using SiRNA PEI transfection reagent (MaoKang, Shanghai, China). The medium was changed 2 days later, and after overnight incubation [58], cells were equilibrated in fresh medium for 2 h, then cells were treated with 1 μg/mL APN for 1 h or 24 h and the supernatants and cells were collected separately for further study.

### 2.7. ELISA

The dissected luteal tissues were homogenized in cold PBS and centrifuged at 5000× *g* for 10 min. The supernatants were collected and the total protein was analyzed using a BCA Protein Assay Kit (Beyotime Biotechnology, Shanghai, China). The concentrations of APN in sera as well as CLs were measured using ELISA (BYabscience, Nanjing, China). The sensitivity was 0.1 ng/mL, with an inter-assay coefficient of variation (CV) of 12% and an intra-assay CV of <8%.

### 2.8. RIA

The P4 levels of sera and media were analyzed using an RIA kit (Bnibt, Beijing, China). The sensitivity was 0.02 ng/mL, with an inter-assay CV of 7.2% and an intra-assay CV of <8.9%.

### 2.9. Statistical Analyses

Data are shown as means ± SEM. A Student’s *t*-test was applied for comparisons of two datasets. A one-way analysis of variance and a Tukey post hoc test were applied for comparisons of multiple datasets. Statistical significance is indicated with asterisks, with *, ** and *** reflecting *p* < 0.05, *p* < 0.01 and *p* < 0.001, respectively. Statistical analyses were conducted in GraphPad Prism v. 6.0 software (GraphPad Software, San Diego, CA, USA).

## 3. Results

### 3.1. Luteal Morphology and Steroidogenesis during the Estrous Cycle in Goat Ovaries

**Figure 1:**
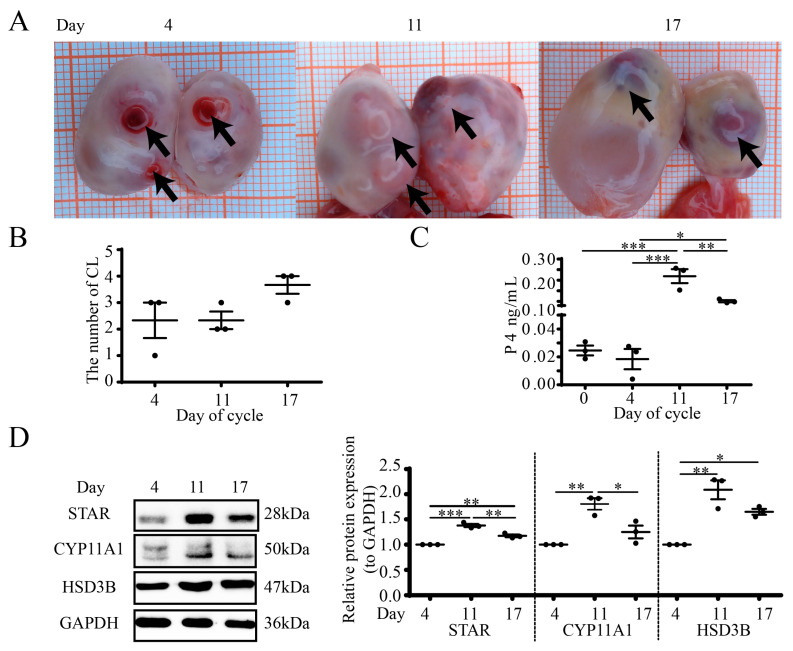
Different luteal stages and steroidogenesis in goat ovaries. (**A**) Photographs of ovaries from different days in the estrous cycle, with arrows indicating individual CLs. (**B**) Each data point is the number of CLs. (**C**) RIA for serum P4. (**D**) WB for steroidogenic proteins (STAR/CYP11A1/HSD3B). Days 0, 4, 11 and 17 represent the days in the estrous cycle, with Day 0 as the onset of estrus. Data are expressed as means ± SEM and the statistical difference was analyzed by a one-way ANOVA followed by a Tukey’s multiple comparisons test. *, ** and *** reflect *p* values of <0.05, *p* < 0.01 and *p* < 0.001, respectively.

The morphology of goat ovaries at D4, D11 and D17 is shown in Figure 1A. The luteal color gradually changed from bright red to dark red, and the number of CLs is indicated in Figure 1B. The serum P4 concentration increased as CLs developed (Figure 1C), with the highest level on D11 (*p* < 0.01). The luteal expression of steroidogenic proteins increased on D11 (STAR, *p* < 0.001; CYP11A1, *p* < 0.01; HSD3B, *p* < 0.01) and D17 (STAR, *p* < 0.01; HSD3B, *p* < 0.05) compared with that on D4 (Figure 1D).

### 3.2. Localization and Structures of Luteal APN during the Estrous Cycle in Goats

**Figure 2:**
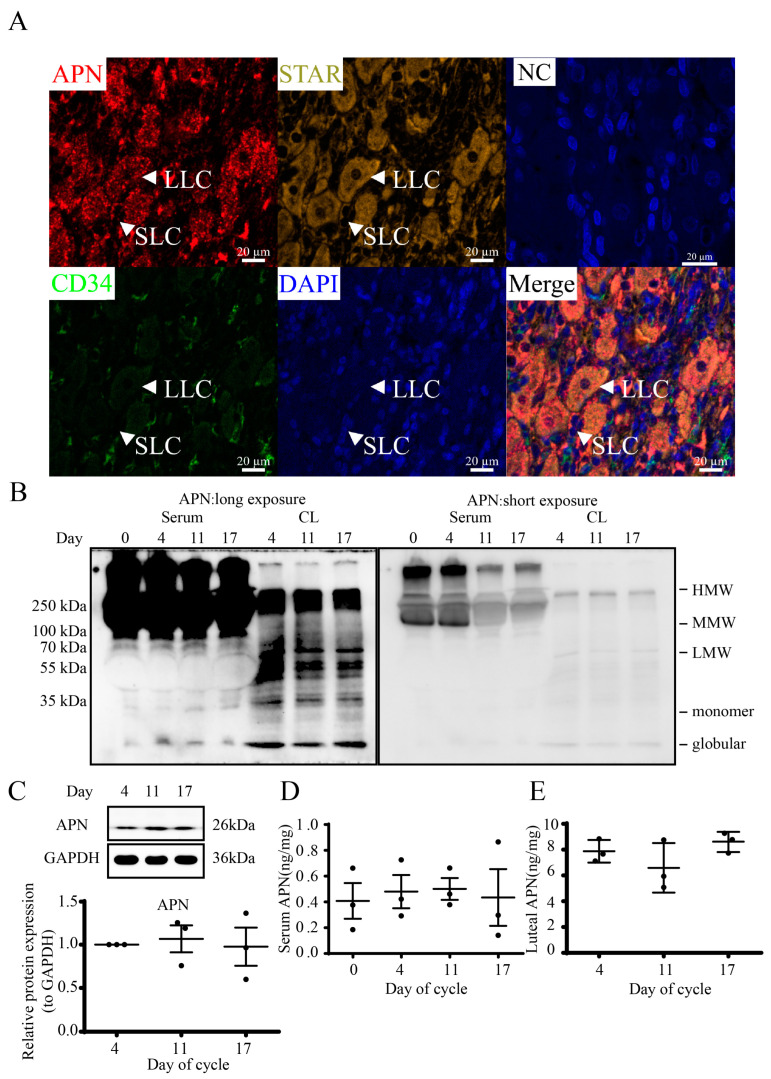
Localization and structures of luteal APN during the estrous cycle in goats. (**A**) Luteal tissue was paraffin-embedded and processed for multiplex immunohistochemistry. APN (dark red), STAR (olive), CD34 (green) and DAPI staining (blue) for nucleus. NC, negative control; SLC, small luteal cells; LLC, large luteal cells. Scale bar = 20 µm. (**B**) Structure of APN in sera and CL. Non-reduced and non-heated conditions were used for SDS-PAGE. HMW, high molecular weight (~360–540 kDa); MMW, middle molecular weight (~180 kDa); LMW, low molecular weight (~90 kDa); monomer (~30 kDa); globular fragment (18~25 kDa). (**C**) WB for the whole luteal APN during the estrous cycle in goats. ELISA for the whole APN in sera (**D**) and CL (**E**) (ng/mg protein). Days 0, 4, 11 and 17 represent the days in the estrous cycle, with day 0 as the onset of estrus. Data are expressed as means ± SEM and the statistical difference was analyzed by a one-way ANOVA followed by a Tukey’s multiple comparisons test.

APN and STAR normally colocalized in luteal cells including small (SLC) and large (LLC) luteal cells in paraffin-embedded tissue (Figure 2A). Most luteal cells were surrounded by blood vessels marked with CD34. Under non-reducing and non-heat-denaturing conditions, separated multimer forms of APN (Figure 2B), including HMW, MMW and LMW APN in sera and CLs and additional monomer and globular APN, were found in CLs (Figure 2B, left, long exposure; Figure 2B, right, short exposure; Supplementary Figure S1). The HMW multimer was expressed in sera more than in CLs, while LMW trimers and monomer and globular APN were expressed in CLs more than in sera. The luteal content of the whole APN was detected by WB and the results showed that there was no difference during the estrous cycle (Figure 2C).

The concentrations of serum and luteal APN during the estrous cycle were also measured by ELISA. Both serum and luteal APN profiles (Figure 2D,E) and structures revealed no significant differences on D4, D11 and D17, and the APN content was about 1 ng/mg in sera and 10 ng/mg (per mg of total protein) in CLs.

### 3.3. APN Inhibits P4 Synthesis and Steroidogenic Protein Expression in Goat Luteal Cells

**Figure 3:**
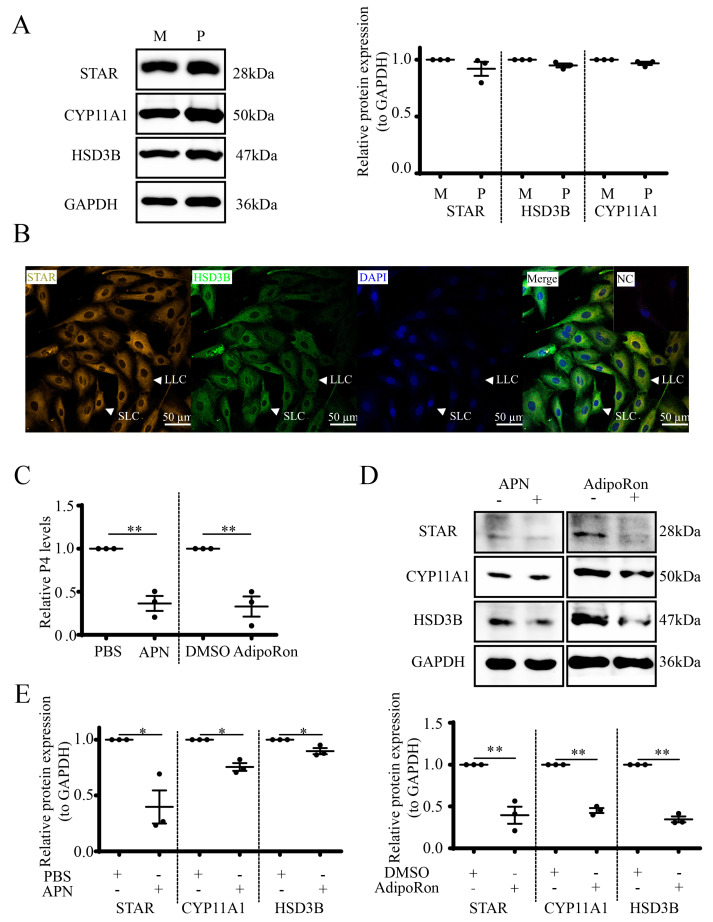
APN/AdipoRon inhibits luteal steroidogenesis. (**A**) WB for CYP11A1, HSD3B and STAR of CLs at middle cycle and pregnancy. M, mid-cycle; P, pregnancy. (**B**) Luteal steroidogenic cells from pregnant goat CLs were identified by mICC. STAR (olive), HSD3B (green) and DAPI (blue). NC, negative control. Scale bar = 50 µm. Luteal cells were treated with 1 μg/mL APN or 25 μM AdipoRon for 24 h, and media were collected to detect P4 concentrations by RIA (**C**) and luteal cell lysates were identified by STAR, CYP11A1 and HSD3B expressions by WB (**D**). Relative STAR, CYP11A1 and HSD3B expression levels were analyzed based on WB (**E**). Data are expressed as means ± SEM and the statistical difference was analyzed by a Student’s *t*-test. * and ** reflect *p* values of <0.05 and *p* < 0.01, respectively.

In order to determine the function of APN on luteal P4 synthesis, we first validated the middle luteal STAR, CYP11A1 and HSD3B expression levels, which were similar to pregnant stage (Figure 3A). Thus, pregnant CLs were used for the subsequent experiments. Luteal cells separated from the pregnant CL were identified by the STAR and HSD3B (Figure 3B) expressions in the cytoplasm. Then, recombinant APN and its receptor agonist AdipoRon were applied to treat the isolated cells, and the results showed that both the P4 concentration (*p* < 0.01, Figure 3C) and the STAR, CYP11A1 and HSD3B levels (Figure 3D,E) decreased at 24 h following treatment with 1 μg/mL APN (*p* < 0.05) or 25 μM AdipoRon (*p* < 0.01).

### 3.4. APN Activates the APPL1-LKB1/AMPK Pathway in Goat Luteal Cells

**Figure 4:**
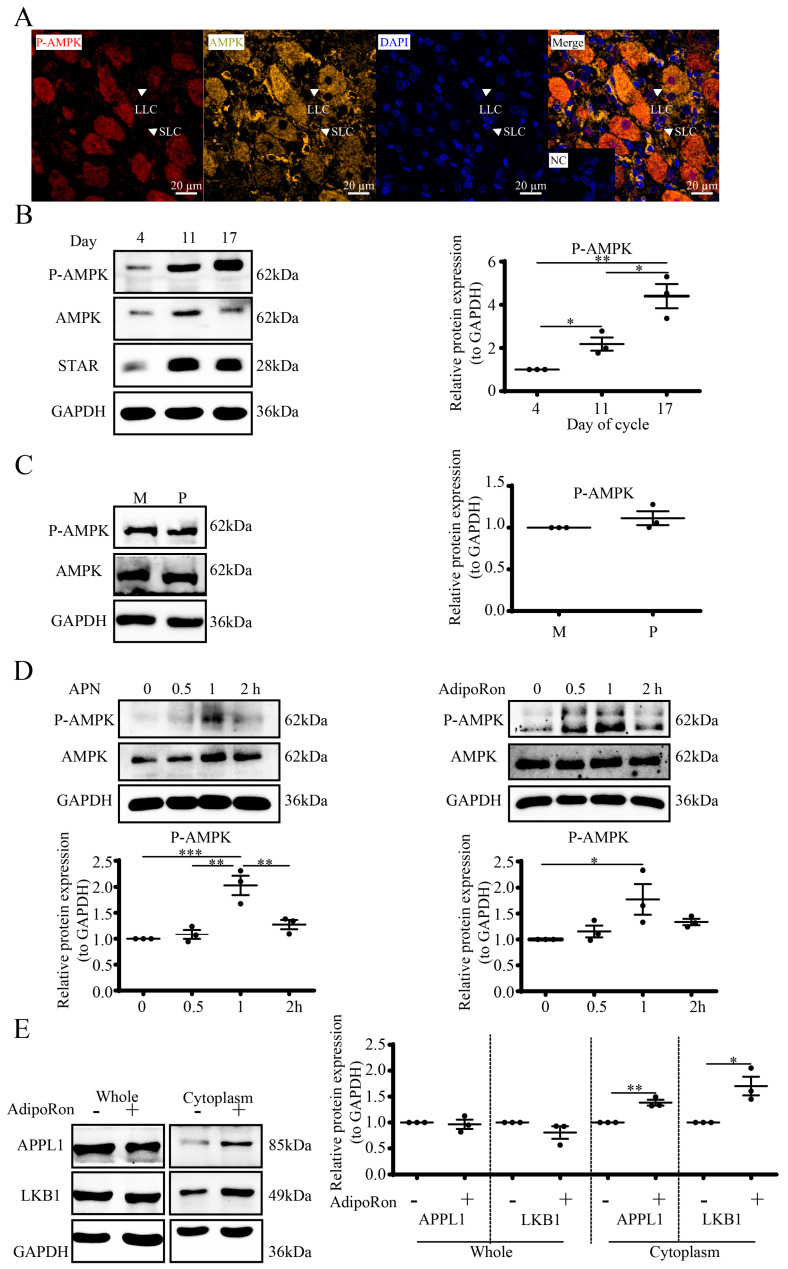
APN activates the AMPK signal in goat luteal steroidogenic cells. (**A**) Luteal tissue was paraffin-embedded and processed for mIHC. P-AMPK (dark red), AMPK (olive) and DAPI staining (blue) for nucleus. NC, negative control; SLC, small luteal cells; LLC, large luteal cells. Scale bar = 20 µm. (**B**) WB for P-AMPK, AMPK and STAR of cyclic CLs. (**C**) WB for P-AMPK in mid-cycle and pregnant CLs. M, mid-cycle; P, pregnancy. (**D**) Cells were treated with 1 μg/mL APN (left) or 25 μM AdipoRon (right) for 0, 0.5, 1 and 2 h to detect P-AMPK by WB. (**E**) Cells were treated with 1 μg/mL APN or 25 μM AdipoRon for 1 h to detect whole and cytoplasmic APPL1 and LKB1 by WB. Days 0, 4, 11 and 17 represent the days in the estrous cycle, with day 0 as the onset of estrus. Data are shown as means ± SEM. A Student’s *t*-test was used for the comparison of two datasets. A one-way analysis of variance was followed by a Tukey’s multiple comparisons test. Statistical significance is indicated with asterisks *, ** and ***, reflecting *p* values of <0.05, *p* < 0.01 and *p* < 0.001, respectively.

To further elucidate the role of APN in P-AMPK signaling in luteal cells, we first detected the cellular location of AMPK and P-AMPK by mIHC, with the results showing that they were colocalized in luteal cells including SLC and LLC, with AMPK scattered in the cytoplasm and P-AMPK in both the nuclei and cytoplasm (Figure 4A).

We also detected P-AMPK expression in goat cyclic CLs by WB. The results (Figure 4B) showed that compared to D4, the expression level of P-AMPK was higher on D11 (*p* < 0.05) together with D17 (*p* < 0.01). Compared with D11, the P-AMPK expression was markedly elevated on D17 (*p* < 0.05). In addition, the mid-cycle luteal P-AMPK expression level was similar to the pregnant stage (Figure 4C).

Next, we used APN and AdipoRon to activate the AMPK signal. Luteal cells were treated with recombinant APN (1 μg/mL) or AdipoRon (25 μM) for 0, 0.5, 1 and 2 h. The WB results showed that APN (*p* < 0.001) along with AdipoRon (*p* < 0.05) increased the phosphorylation of AMPK at the threonine 172 site after 1 h (Figure 4D). Although the global protein levels (Figure 4E) of both APPL1 and LKB1 did not change after AdipoRon treatment, the cytoplasmic APPL1 (*p* < 0.01) together with LKB1 (*p* < 0.01) obviously increased.

### 3.5. AdipoRon Regulates P4 by AMPK in Goat Luteal Cells

**Figure 5:**
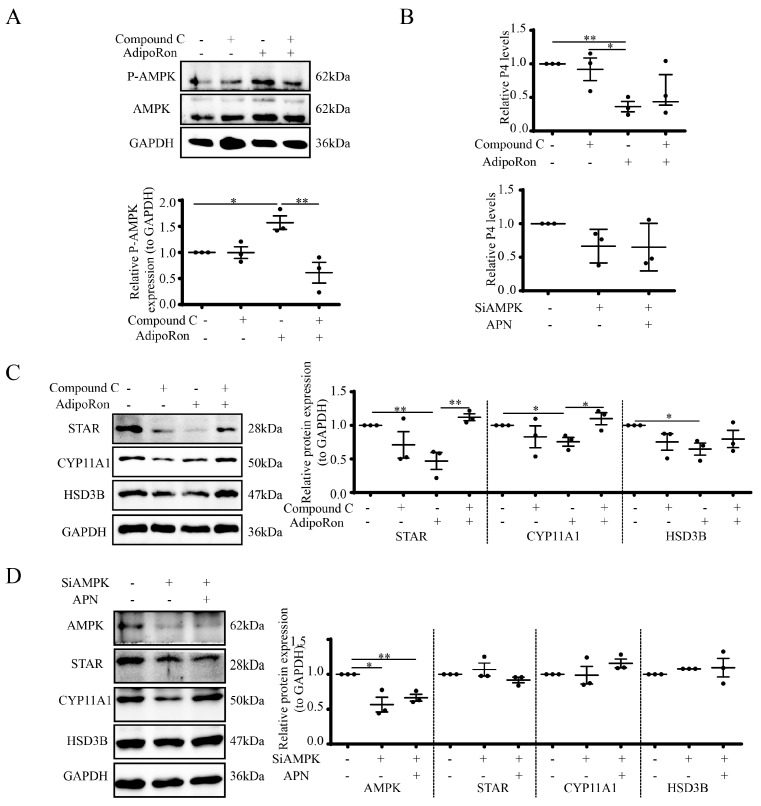
APN/AdipoRon modulates steroidogenesis through the AMPK signal in goat luteal cells. (**A**) Luteal cells were pretreated with Compound C (10 μM, 1 h) prior to treatment with AdipoRon (25 μM, 1 h), and cell lysates were processed to detect P-AMPK by WB. Luteal cells were pretreated with Compound C (10 μM, 1 h) prior to treatment with AdipoRon (25 μM, 24 h), media were collected to detect the P4 concentration by RIA (**B**, upper) and cell lysates were processed to detect STAR, CYP11A1 and HSD3B by WB (**C**). (**D**) Luteal cells were pretreated with SiAMPK for 48 h, then cells were treated with 1 μg/mL APN for 24 h to detect AMPK, STAR, CYP11A1 and HSD3B by WB, and media were collected to detect the P4 concentration by RIA (**B**, lower). SiAMPK, SiRNA for AMPK; scrambled non-targeting RNA was transfected into cells as a negative control. Data are expressed as means ± SEM and the statistical difference was analyzed by a one-way ANOVA followed by a Tukey’s multiple comparisons test. * and ** reflect *p* values of <0.05 and *p* < 0.01, respectively.

To investigate whether AMPK regulates luteal steroidogenesis, cells were incubated with Compound C (10 μM, inhibitor of P-AMPK) for 1 h, following by treatment with 25 μM AdipoRon for 1 h to detect the P-AMPK expression and 24 h to detect the steroidogenic protein expression. The results showed that AdipoRon induced P-AMPK expression (*p* < 0.05), and Compound C inhibited the AdipoRon-induced P-AMPK expression (*p* < 0.01, Figure 5A). Treatment with 25 μM AdipoRon for 24 h reduced the P4 release (*p* < 0.01). Although Compound C did not reverse the AdipoRon-reduced P4 release (Figure 5B upper), it reversed the AdipoRon-induced repression of steroidogenic proteins, including STAR (*p* < 0.01) and CYP11A1 (*p* < 0.05) (Figure 5C). For additional verification of the involvement of AMPK in APN-mediated steroidogenesis, we also suppressed AMPK by SiRNA (*p* < 0.05) and found that APN treatment failed to affect the P4 release (Figure 5B lower) or STAR, CYP11A1 and HSD3B expressions (Figure 5D).

### 3.6. Expression and Localization of APN Receptors in Goat CLs

**Figure 6:**
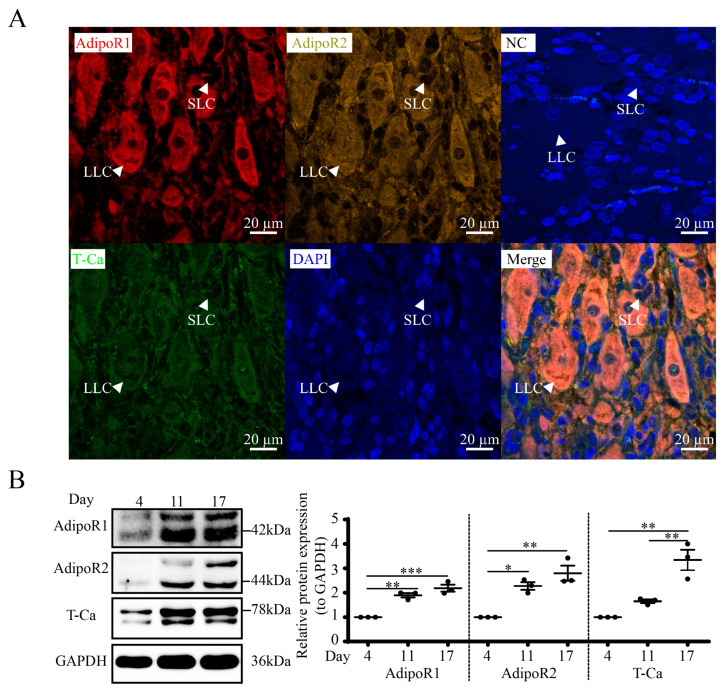
Expression profiles of APN receptors in goat CLs. (**A**) Mid-cycle luteal tissue was paraffin-embedded and processed for mIHC. AdipoR1 (dark red), AdipoR2 (olive), T-Ca (green) and DAPI (blue). NC, negative control; SLC, small luteal cells; LLC, large luteal cells. Scale bar = 20 µm. (**B**) WB for AdipoR1, AdipoR2 and T-Ca of CLs at different stages of the estrous cycle. Days 0, 4, 11 and 17 represent the days in the estrous cycle, with day 0 as the onset of estrus. Data are expressed as means ± SEM and the statistical difference was analyzed by a one-way ANOVA followed by a Tukey’s multiple comparisons test. *, ** and *** reflect *p* values of <0.05, *p* < 0.01 and *p* < 0.001, respectively.

We found that AdipoR1, AdipoR2 and T-Ca colocalized in luteal cells, including SLC and LLC, from mIHC (Figure 6A). The WB results (Figure 6B) showed that luteal AdipoR1, AdipoR2 and T-Ca levels are related to the estrous cycle. Compared to D4, the expressions of APN receptors were vastly higher on D11 (AdipoR1, *p* < 0.01; AdipoR2, *p* < 0.05) and D17 (AdipoR1, *p* < 0.001; AdipoR2, *p* < 0.01; T-Ca, *p* < 0.01).

### 3.7. APN Increases P-AMPK through AdipoR2 in Goat Luteal Cells

**Figure 7:**
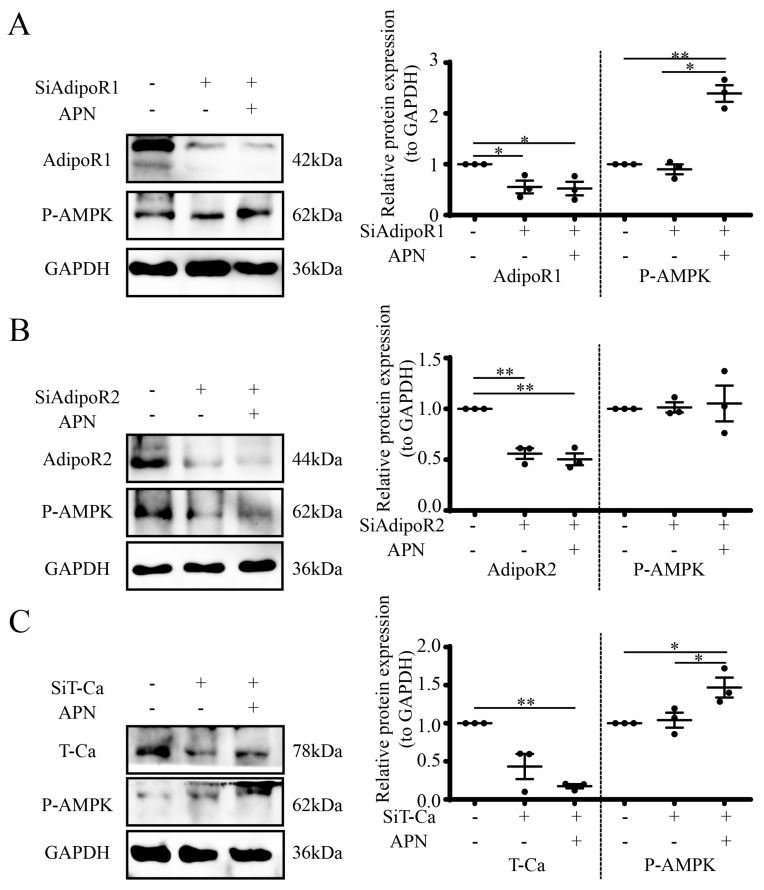
APN increases P-AMPK through AdipoR2. Luteal cells were pretreated with SiAdipoR1 (**A**), SiAdipoR2 (**B**) or SiT-Ca (**C**) for 48 h, then cells were treated with 1 μg/mL APN for 1 h to detect target receptors and P-AMPK by WB (**A**–**C**). SiAdipoR1: SiRNA for AdipoR1; SiAdipoR2, SiRNA for AdipoR2; SiT-Ca, SiRNA for T-Ca; scrambled non-targeting RNA was transfected into cells as a negative control. Data are expressed as means ± SEM and the statistical difference was analyzed by a one-way ANOVA followed by a Tukey’s multiple comparisons test. * and ** reflect *p* values of <0.05 and *p* < 0.01, respectively.

To explore the function of APN and its receptors in the control of luteal P-AMPK, luteal cells were first transfected with SiRNA (100 μM), and 48 h later, APN (1 μg/mL) was added to luteal cells for 1h to detect P-AMPK. The results showed that after the suppression of AdipoR1 by SiRNA (*p* < 0.05), APN treatment enhanced P-AMPK expression (*p* < 0.05, Figure 7A). However, after the suppression of AdipoR2 by SiRNA (*p* < 0.01), APN treatment failed to affect the increase in the P-AMPK expression (Figure 7B). The suppression of T-Ca by SiRNA (*p* < 0.05) achieved a similar result to AdipoR1 (Figure 7C).

### 3.8. APN Inhibits Steroidogenesis through AdipoR2 in Goat Luteal Cells

**Figure 8:**
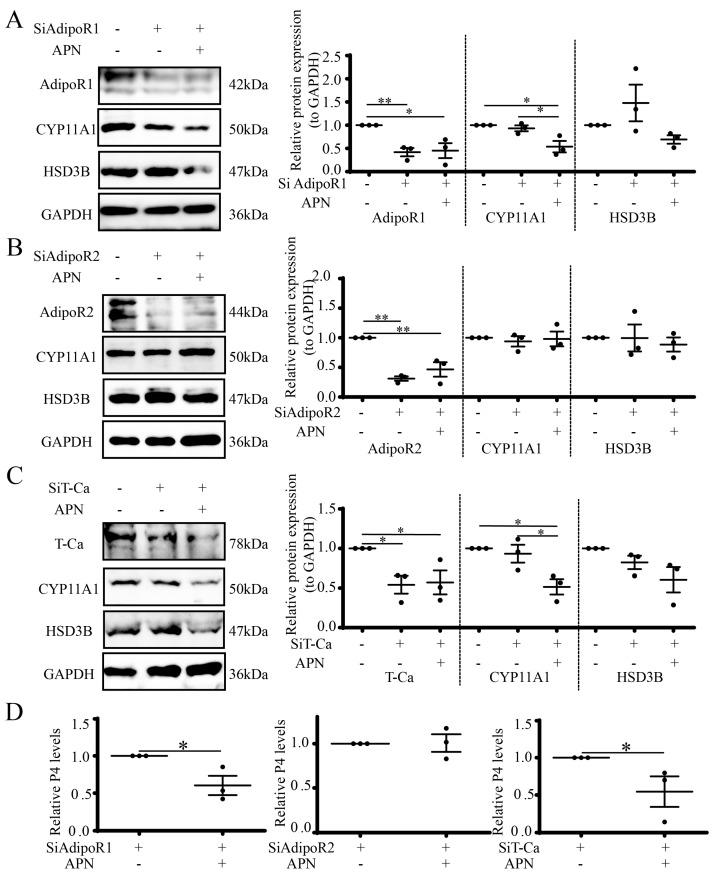
APN decreases steroidogenesis through AdipoR2. Luteal cells were pretreated with SiAdipoR1 (**A**), SiAdipoR2 (**B**) or SiT-Ca (**C**) for 48 h, then cells were treated with 1 μg/mL APN for 24 h to detect target receptors, CYP11A1 and HSD3B by WB (**A**–**C**) and media were collected to detect the P4 concentration by RIA and the levels were normalized to the control (**D**). SiAdipoR1: SiRNA for AdipoR1; SiAdipoR2, SiRNA for AdipoR2; SiT-Ca, SiRNA for T-Ca. Scrambled non-targeting RNA was transfected into cells as a negative control. Data are expressed as means ± SEM and the statistical difference was analyzed by a one-way ANOVA followed by a Tukey’s multiple comparisons test. * and ** reflect *p* values of <0.05 and *p* < 0.01, respectively.

To investigate the function of APN and its receptors in the control of luteal steroidogenesis, luteal cells were first transfected with SiRNA (100 μM), and 48 h later, APN (1 μg/mL) was added to luteal cells for 24 h to detect steroidogenic proteins and the P4 concentration. The results showed that after the suppression of AdipoR1 by SiRNA (*p* < 0.05), APN treatment reduced the CYP11A1 expression (*p* < 0.05) but not the HSD3B expression (Figure 8A). However, after the suppression of AdipoR2 by SiRNA (*p* < 0.01), APN treatment failed to affect the CYP11A1 and HSD3B expressions (Figure 8B). The suppression of T-Ca by SiRNA (*p* < 0.05) achieved a similar result to AdipoR1 (Figure 8C). The P4 concentration exhibited similar results to the CYP11A1 expression (*p* < 0.05) (Figure 8D).

## 4. Discussion

The current study first identified the structure and expression profiles of APN and its receptors in goat serum and CL, and then applied isolated luteal steroidogenic cells to explore the role of APN and the mechanisms of regulation of luteal steroidogenesis in goats. We found that APN inhibited luteal steroidogenesis through the AdipoR2/AMPK pathway in goat luteal cells, although AdipoR1 and T-Ca showed similar expression patterns to AdipoR2 in goat CLs.

APN includes globular and full-length forms, which produce different effects in various tissues. Previous findings have revealed different isoforms of APN in circulation and in specific tissues [20,52,59,60,61]. Globular APN binds more cell membranes than full-length APN in mice C2C12 myocytes and skeletal muscle cells, which is in contrast to what occurs in hepatocytes [62]. AdipoR1 and AdipoR2 show dissimilar distribution patterns and binding affinities with globular and full-length APN in mouse C2C12 myocytes [62], and APN contains full-length isoforms in porcine sera [63]. The current high HMW APN content in sera and the LMW, monomeric and globular APN in CL indicate that full-length APN accounts for the largest fraction in goat CL and exerts an important role in CLs. Studies in rodents and humans have showed that trimeric and hexametric APN is conveyed from the serum into cerebrospinal fluid to regulate various physiological functions in the brain [64]. HMW APN is associated with diabetes pathogenesis [65] and is vasoprotective [66]. MMW and LMW APN is delivered into the brain to control dietary intake [20]. Globular APN may reverse osteoporosis in postmenopausal women [67]. These studies suggest that the multiple APN forms have distinct functions in different tissues. The present results demonstrate HMW APN in sera as a stock solution might be decomposed into MMW or LMW APN, which binds to APN receptors on luteal cells to regulate steroidogenesis. In addition, APN structures in serum and CL are independent of the estrous cycle or pregnancy. Therefore, we used full-length APN to treat luteal cells in the present study.

The current concentrations of APN in goat sera and CLs are also independent of the estrous cycle (Figure 2B and Supplementary Figure S1), which is not consistent with previous studies of the CLs of dairy cow and buffalos [12,14] and previous studies of porcine serum, which showed that the APN concentration varies during the estrous cycle [63]. However, our findings are compatible with the studies of sera APN levels in women [68,69]. This is most likely caused by the different affinities of APN for receptors in different species.

Numerous studies have shown that APN is localized in the oocyte, granulosa cells, theca cells and luteal cells in various species [16,70,71], indicating APN might be involved in the control of follicular and luteal development. The localization of APN in luteal steroidogenic cells marked with STAR and less so in vessels marked with CD34, along with the APN/AdipoRon-induced decreases in P4 secretion and steroidogenic protein (STAR/CYP11A1/HSD3B) expression in isolated luteal cells, indicates that APN is involved in regulating goat luteal function. These results are consistent with prior studies showing that APN inhibited P4 production and the gene expressions of STAR, CYP11A1 and HSD3B in cultured luteal cells of buffalos [12,72]. APN also exhibits vasoprotective effects, and low APN levels correlate with vascular damage within the ischemia [73], showing that APN might play a role in the control of luteal angiogenesis.

APN receptors have been found to be expressed in mammalian ovaries [12,74]. AdipoR1/2 was primarily localized in theca cells, oocytes and CLs and less so in granulosa cells of rat ovaries [70]. In goat ovaries, AdipoR1/2 has been reported in oocytes, cumulus cells and granulosa cells [11]. However, there are no reports about the expression of T-Ca in ovaries. The current findings of AdipoR1/2 in goat luteal steroidogenic cells with the colocalization of STAR is consistent with Gupta’s study in buffalos [12], indicating that APN might regulate the luteal function by binding to AdipoR1 and AdipoR2 in goats. The increased AdipoR1/2 expression level with the luteal phase is consistent with Tabandeh’s study in dairy cows [75]. However, the higher expression of AdipoR1/2 in mid-cycle and late-cycle CLs is inconsistent with Gupta’s study in buffalo CLs, which showed that AdipoR1/2 was highly expressed in the early cycle and regressing CLs and poorly expressed in mid- and late-cycle CLs [12]. Studies have shown that a higher level of AdipoR1 mRNA expression was found in porcine granulosa cells rather than in CLs and theca cells across the estrous cycle, while the lowest level was observed in regressing CLs. The highest AdipoR2 gene expression emerged in mature CLs, while the lowest was noted in theca cells [63]. These observations revealed that the expression patterns of AdipoR1 and AdipoR2 vary within and across species.

APN can activate AMPK, MAPK, PPAR-α, mTOR, PI3K/Akt, STAT3 and NF-κB in multiple tissues, including liver, muscle and ovary tissues, to play various biological roles [76,77,78,79,80], and most biological processes of APN are mediated by the AMPK signal. APN mediates the phosphorylation of AMPK in an APPL1-dependent or -independent manner [33,81,82]. AMPK can be activated by both globular and full-length APN in skeletal muscles, while it can be activated primarily by full-length APN in the liver [62]. AdipoRon, an agonist of APN, activates AMPK signaling in multiple tissues, including mice muscles and liver [83,84] and human luteinized granulosa cells [51]. As expected, both APN and AdipoRon treatments increased P-AMPK in goat luteal cells, and LKB1 and AMPK were both colocalized in luteal cells (not presented here). It is widely known that under energy stress, LKB1 is the key upstream kinase of the energy sensor AMPK [85]. The current finding that AdipoRon enhanced the translocation of cytosolic APPL1 and LKB1 and the activation of AMPK in goat luteal cells fits with a previous study [33], indicating that the regulation of AMPK activity could be tuned in accordance with the surrounding hormones and nutritional requirements of the CL.

The presence of AMPK has been found in a variety of ovarian cells of several mammalian species [86,87,88]. AICAR, an AMPK agonist, was reported to increase STAR/P450scc in adrenal zona fasciculata but decrease STAR/P450scc in adrenal zona reticularis [89]. AdipoRon can activate the AMPK signaling pathway and reduce steroid accumulation in human luteinized granulosa cells [51]. The current result that Compound C reversed the AdipoRon-induced repression of steroidogenic proteins (STAR/CYP11A1/HSD3B) without P4 secretion might indicate that Compound C can regulate not only AMPK but also ALK and participate in autophagy processes [90]. Therefore, to further verify the function of AMPK in the control of steroidogenesis, we suppressed AMPK expression by SiAMPK. After that, APN decreased luteal steroidogenesis by increasing the P-AMPK expression. This finding is in agreement with a previous report that pharmacological stimulation with AICAR or metformin inhibited the secretion of P4 and the expression of STAR, HSD3B and CYP11A1 in bovine luteal cells [91]. Further exploration should be directed to the specific regulatory mechanisms.

The current findings of APN-induced AMPK activation and a decrease in the CYP11A1 expression after suppression of AdipoR1 (not AdipoR2) suggest that AdipoR1 is not sufficient to affect steroid biosynthesis through the AMPK signal. Perhaps higher APN concentrations are necessary for a more detailed analysis and AdipoR2 is required to regulate steroid biosynthesis through the AMPK signal, which further confirm the different functions of AdipoR1 and AdipoR2 in goat luteal cells as in most tissues or cells. However, the findings are not consistent with others; AdipoR1 mainly stimulated AMPK [92] while AdipoR2 mainly stimulated the PPAR pathway [93,94]. Although we did not detect a PPAR signal, the current results demonstrate that AdipoR2 contributes substantially more than AdipoR1 to AMPK response and steroidogenesis. It was reported that APN and AdipoRon protected rats and mice from neuron injuries through AdipoR1-AMPK [52,95,96]. Resveratrol alleviated the oxidative stress in muscle fibers via the AdipoR1-AMPK-PGC-1α pathway in pigs [92], while esculetin activated the AdiopR2-AMPK pathway, which was beneficial for hepatosteatosis and insulin resistance in HFD-induced obese mice [97]. The analgesic efficacy of acupuncture in the spinal cord was at least in part due to the APN/AdipoR2-AMPK pathway [98]. Consequently, the APN/AdipoR2-AMPK pathway is viable in specific tissues, and these findings clarify the important regulatory mechanism of AdipoR2, suggesting that the activation of AdipoR1/2-AMPK is species or tissue specific.

The current findings of colocalization of T-Ca with STAR in goat CLs and T-Ca alteration during different luteal phases suggest that APN may contribute to the luteal function through T-Ca. Studies have shown that the APN-induced T-Ca complex may engage in AMPK signaling [26] and that T-Ca selectively binds to HMW APN with a high affinity but not trimeric or globular forms [99]. T-Ca is abundantly present in endothelial cells, pericytes, smooth muscle and skeletal muscle [100,101,102], and T-Ca knockout mice exhibited an increase in blood APN and a decrease in the contact of APN with endothelial cells in vascular tissues [103]. Furthermore, T-Ca is a requisite component for the cardioprotective and regenerative functions of APN [102]. Our results showing APN-induced AMPK activation and decreases in CYP11A1 expression and P4 levels after T-Ca suppression suggest that APN/T-Ca interaction may not contribute to luteal steroidogenesis. Nevertheless, it might have other critical biological functions, such as luteal revascularization, which is also important for the luteal function in goat ovaries.

APN receptors display a differential affinity for multiple APN forms. AdipoR1 seems to be involved in KGN survival via P-Bad [104]. T-Ca promotes islet proliferation via upregulating the Notch pathway [27]. Whether Bad or Notch pathways regulate goat luteal cell survival by AdipoR1 or T-Ca has not been addressed, and this needs further investigation.

## 5. Conclusions

In conclusion, the APN structure and content are similar in different luteal phases, with far more full-length APN than globular APN both in CLs and sera. The structural differences in APN may lead to functional differences in CLs and sera. APN and its receptors are predominantly located in luteal steroidogenic cells, with increased expressions during middle and late luteal periods. APN and AdipoRon reduce luteal steroidogenesis through the AdipoR2/AMPK pathway.

## Figures and Tables

**Table 1:** Details of antibodies.

Antibodies	Cat No.	MFRS	Antigen Source	Host	Dilution
STAR	A1035	ABclonal	Human	Rabbit	WB: 1:1000; mIHC: 1:100
HSD3B	A1823	ABclonal	Human	Rabbit	WB: 1:1000; mIHC: 1:100
CYP11A1	A1713	ABclonal	Human	Rabbit	WB: 1:1000
APN	A2543	ABclonal	Human	Rabbit	WB: 1:1000; mIHC: 1:200
T-Ca	A1761	ABclonal	Human	Rabbit	WB: 1:1000; mIHC: 1:200
AdipoR1	A1509	ABclonal	Human	Rabbit	WB: 1:2000; mIHC: 1:100
AdipoR2	A12777	ABclonal	Human	Rabbit	WB: 1:2000; mIHC: 1:100
APPL1	A4606	ABclonal	Human	Rabbit	WB: 1:2000
AMPK	A7339	ABclonal	Human	Rabbit	WB:1:2000; mIHC: 1:100
P-AMPK	AP0116	ABclonal	Human	Rabbit	WB:1:2000; mIHC: 1:100
GAPDH	T0004	Affinity	Human	Mouse	WB: 1:5000
LKB1	A2122	ABclonal	Human	Rabbit	WB:1:2000
HRP Anti-Mouse IgG	AS003	Abclonal	Mouse	Goat	WB: 1:20,000
HRP Anti-Rabbit IgG	AS014	Abclonal	Rabbit	Goat	WB: 1:20,000

## Data Availability

The data underlying this article will be shared upon reasonable request to the corresponding author.

## Supplementary Materials

The following supporting information can be downloaded at: https://www.mdpi.com/article/10.3390/cells12101393/s1, Figure S1: Serum and luteal APN structures at different stages of estrous cycle and AdipoR1 localization in goats.

## Abbreviations

Adiponectin (APN); adenosine monophosphate-activated protein kinase (AMPK); adaptor protein, phosphotyrosine interacting with PH domain and leucine zipper 1 (APPL1); corpus luteum (CL); cytochrome P450 cholesterol side-chain cleavage enzyme (CYP11A1); high molecular weight (HMW); 3-beta-hydroxysteroid dehydrogenase (HSD3B); low molecular weight (LMW); liver kinase B1 (LKB1); multiplex immunohistochemistry/immunocytochemistry (mIHC/mICC); middle molecular weight (MMW); progesterone (P4); peroxisome proliferator-activated receptor (PPAR); steroidogenic acute regulatory protein (STAR); T-cadherin (T-Ca); sodium dodecyl sulfate-polyacrylamide gel electrophoresis (SDS-PAGE).
