# Supplementary material for: APN Expression in Serum and Corpus Luteum: Regulation of Luteal Steroidogenesis Is Possibly Dependent on the AdipoR2/AMPK Pathway in Goats
Source: Cells. 2023 May 15;12(10):1393. doi: 10.3390/cells12101393 (PMC10217118; doi:10.3390/cells12101393)
Supplement: Supplementary file 1 [file cells-12-01393-s001.zip › cells-2336045-supplementary.pdf]

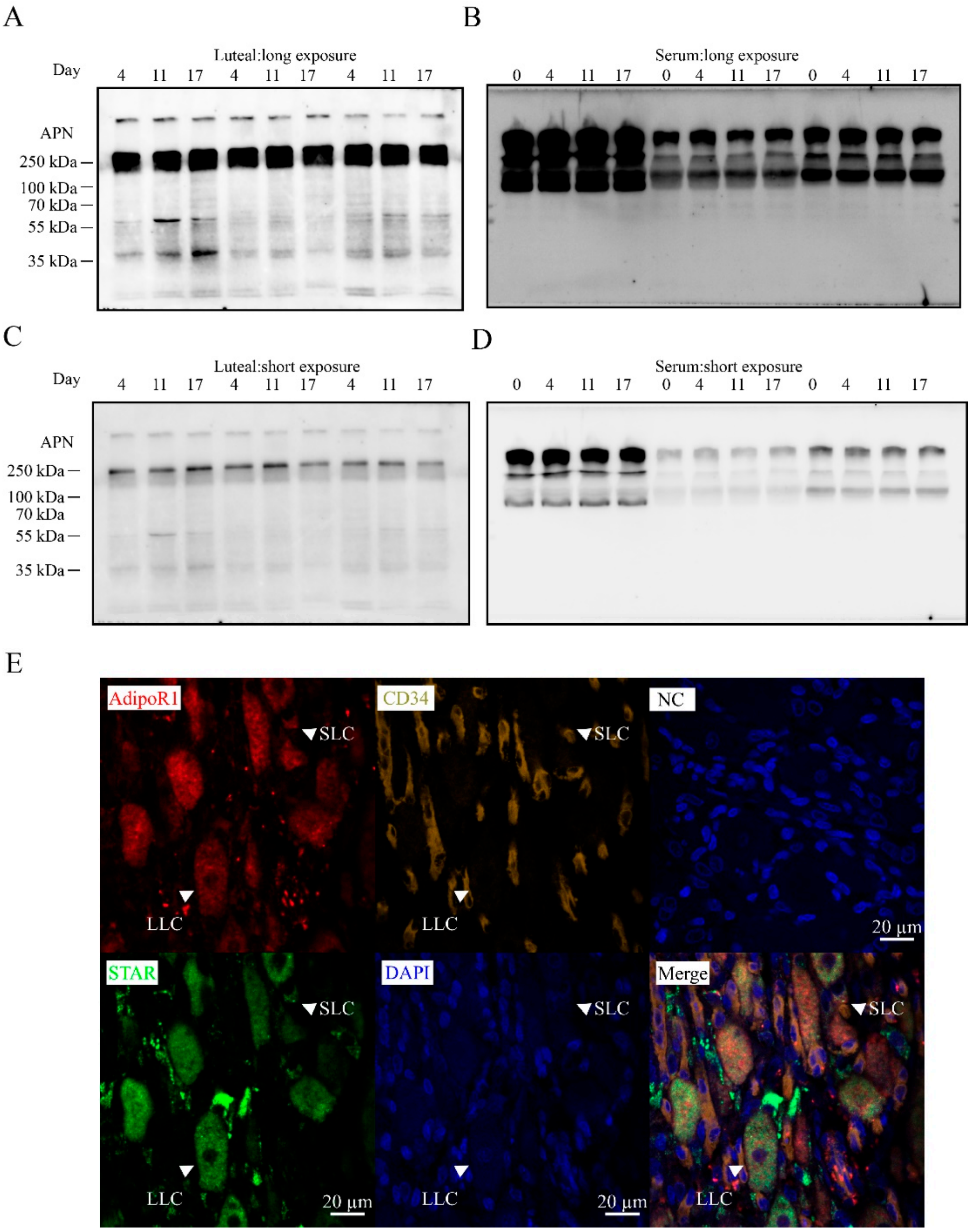


**Supplementary Figure S1** Serum and luteal APN structures at different stages of estrus cycle and AdipoR1 localization in goats. (A-D) Related to Fig. 2 Serum and luteal APN structures at different stages of estrus cycle in goats. WB with nonreduced and nonheated SDS-PAGE long exposure for APN in luteal tissue (A) and serum (B), short exposure for APN in luteal tissue (C) and serum (D); (E)Related to Fig. 6 Expression profile of AdipoR1 in goat CL. The luteal tissue was paraffin-embedded and processed for mIHC. Days 0, 4, 11 and 17 represent the days in the estrous cycle, with day 0 as the onset of estrus. Shown are AdipoR1 (Dark red), CD34 (Olive), STAR (Green) and DAPI (Blue). NC, negative control; SLC, small luteal cells; LLC, large luteal cells. Scale bar=20 μm.
